# In Vitro and In Vivo Evaluation of *Pediococcus acidilactici* Pedio6-1 for Purine Metabolism Modulation in Diet-Induced Obese Mice

**DOI:** 10.3390/microorganisms14081677

**Published:** 2026-07-30

**Authors:** Haohua Fu, Hengjia Ni, Jianhui Wang, Tuo Leng, Shusong Wu, Pan Huang, Jianjun Li, Cimin Long, Yulong Yin

**Affiliations:** 1 School of Food Science and Bioengineering, Changsha University of Science and Technology, Changsha 410114, China; rayaiyz@163.com (H.F.); wangjh0909@163.com (J.W.); lengtuo95@163.com (T.L.); 2 College of Animal Science and Technology, Hunan Agriculture University, Changsha 410125, China; wush688@hunau.edu.cn; 3 Key Laboratory of Agro-Ecological Processes in Subtropical Region, Hunan Provincial Key Laboratory of Animal Nutritional Physiology and Metabolic Process, Hunan Research Center of Livestock and Poultry Sciences, South Central Experimental Station of Animal Nutrition and Feed Science in the Ministry of Agriculture, Institute of Subtropical Agriculture, Chinese Academy of Sciences, Changsha 410125, China; huangpan1990@isa.ac.cn (P.H.); jianjunli@isa.ac.cn (J.L.); lcm@isa.ac.cn (C.L.)

**Keywords:** *Pediococcus acidilactici*, purine metabolism, intestinal microorganisms

## Abstract

Gut lactic acid bacteria are emerging as potential targets for modulating host purine metabolism and alleviating hyperuricemia-related disorders. This study aimed to isolate purine-degrading lactic acid bacterial strains from porcine intestine and systematically evaluate their probiotic potential through both in vitro and in vivo approaches. A strain designated Pedio6-1 was isolated and identified as *Pediococcus acidilactici* based on 16S rRNA sequencing. In vitro assays demonstrated that *P. acidilactici* Pedio6-1 exhibited nearly complete adenine clearance (approaching 100%) and a total purine clearance rate of 30.73%, along with strong tolerance to acidic conditions, bile salts, and gastrointestinal enzymes. To further assess its in vivo efficacy, a high-fat diet-induced obese mouse model was employed. After 8 weeks of intervention, Pedio6-1 supplementation significantly reduced the final body weight and liver index, and markedly decreased hepatic guanine levels (*p* < 0.05), with a trend toward lower total purine content compared to the obese control group. Mechanistically, Pedio6-1 treatment significantly downregulated the hepatic mRNA expression of pro-inflammatory cytokines IL-1β and TLR4 (*p* < 0.05). Serum untargeted metabolomics revealed that Pedio6-1 treatment shifted the metabolic profile toward that of normal diet-fed mice, with differential pathways predominantly enriched in porphyrin metabolism and amino acid biosynthesis and metabolism. Notably, key metabolites with antioxidant and metabolic regulatory functions, including bilirubin, biliverdin, glutathione, citrate, L-cystathionine, and 4-pyridoxic acid, were significantly elevated following treatment, while N-acetylornithine and methylmalonate ester were decreased, indicating coordinated remodeling of oxidative stress defense, vitamin B6 homeostasis, and energy metabolism. Collectively, these findings indicate that Pedio6-1 not only possesses direct purine-degrading activity in vitro, but also ameliorates purine metabolic disturbances, inflammation, and oxidative stress in obese mice, likely through the modulation of porphyrin and amino acid metabolic pathways, highlighting its promise as a functional probiotic candidate for managing obesity-associated purine metabolic disorders.

## 1. Introduction

With the continuous changes in lifestyle and dietary patterns, the intake of purine rich foods has markedly increased, leading to disturbances in purine metabolism in both humans and companion animals such as dogs and cats. This metabolic imbalance can subsequently result in hyperuricemia, gout, and urate urolithiasis [1]. Uric acid is the final product of purine metabolism, and excessive production or impaired excretion of uric acid leads to persistent hyperuricemia, which in severe cases may cause joint inflammation, renal injury, and other systemic complications [2]. In veterinary practice, particularly in breeds predisposed to hyperuricemia such as Dalmatians, abnormalities in purine metabolism have become a critical factor affecting quality of life [3]. Therefore, identifying safe and effective strategies to regulate purine metabolism is of considerable importance for both public health and clinical applications.

Notably, purine metabolic disorders are closely associated with obesity. Obesity not only induces insulin resistance, chronic inflammation, and lipid metabolic dysfunction, but also exacerbates purine metabolic disturbances by impairing hepatic and intestinal functions [4]. Previous studies have demonstrated that serum uric acid levels are significantly elevated in obese individuals, including both humans and experimental animals, and that hyperuricemia is positively correlated with obesity related metabolic syndrome [5]. In high fat diet-induced obese mouse models, hepatic levels of guanine, xanthine, and total purines are significantly increased, indicating a pronounced disruption of purine metabolism under obese conditions [6]. Thus, modulating purine metabolism in obese individuals represents a promising strategy for improving overall metabolic health.

In our previous work, we used Daweizi pigs, a typical fatty type pig breed, as the experimental model and found that pigs fed a low protein diet without soybean meal exhibited reduced fat deposition and increased lean meat percentage. Notably, the total purine content in the longissimus dorsi muscle was significantly decreased to approximately half of that observed in the control group. Further analysis of the colonic microbiota revealed a significant increase in the relative abundance of lactobacilli and a decrease in the Firmicutes to Bacteroidetes ratio in the low protein diet group, suggesting that intestinal lactic acid bacteria may participate in the regulation of host purine metabolism [7].

Previous studies have confirmed that certain strains of lactobacilli and pediococcus possess the capacity to degrade purine nucleosides [8,]. For example, some lactic acid bacteria can efficiently remove adenine, guanine, and hypoxanthine, thereby reducing the intestinal absorption of purines and alleviating the purine burden in the host [9]. These microorganisms directly metabolize purine compounds through their intrinsic purine metabolic pathways and exhibit considerable potential as functional probiotics for regulating uric acid metabolism.

Based on this background, the present study aimed to isolate potential purine-degrading lactic acid bacterial strains from the colonic digesta of Daweizi pigs with relatively low endogenous purine levels. A high-fat diet-induced obese mouse model was used to systematically evaluate the effects of the strain on host purine metabolism, serum metabolomics, and gut microbiota composition. This study is expected to provide a theoretical basis for the development of functional probiotic preparations targeting purine metabolism and offer new insights into the intervention of purine metabolism-related disorders in both humans and companion animals.

## 2. Materials and Methods

### 2.1. Isolation and Purification of Bacterial Strains

Colonic contents were collected from Daweizi pigs fed a low protein diet without soybean meal. The samples were serially diluted tenfold with sterile saline and thoroughly mixed by vortexing to obtain dilutions of 10^−5^, 10^−6^, 10^−7^, and 10^−8^. An aliquot of 100 μL from each dilution was spread onto MRS agar plates (Qingdao Hope Bio Technology Co., Ltd., Qingdao, China) with three replicates prepared for each dilution. The plates were incubated under anaerobic conditions at 37 °C for 72 h. Single colonies were selected based on differences in morphology, color, and size, and were purified by repeated streaking two to three times until pure cultures were obtained. The purified isolates were then inoculated into MRS broth and incubated at 37 °C for 18 to 24 h. The cultures were collected and mixed with 60% glycerol at a volume ratio of 1:1, and then stored at minus 80 °C for further use [10].

### 2.2. Molecular Identification of the Isolates

Genomic DNA of the isolated strains was extracted using a bacterial genomic DNA extraction kit (Tiangen Biotech Co., Ltd., Beijing, China), following the manufacturer’s instructions. The extracted DNA was used as a template for amplification of the 16S rRNA gene using universal primers 27F with the sequence 5′-AGAGTTTGATCCTGGCTCAG-3′ and 1492R with the sequence 5′-GGTTACCTTGTTACGACTT-3′. The polymerase chain reaction system with a total volume of 25 μL contained 12.5 μL Premix Taq (Takara Bio Inc., Kusatsu, Japan), 1 μL DNA template, 0.5 μL of each forward and reverse primer, and double distilled water to the final volume. The amplification conditions were as follows. Initial denaturation at 94 °C for 5 min, followed by 30 cycles of denaturation at 94 °C for 30 s, annealing at 55 °C for 30 s, and extension at 72 °C for 90 s, with a final extension at 72 °C for 10 min. The amplification products were examined by electrophoresis on a 1% agarose gel. Qualified products were sent to Sangon Biotech Co., Ltd. (Shanghai, China) for sequencing. The obtained sequences were compared with those in the NCBI database using BLAST (https://blast.ncbi.nlm.nih.gov/Blast.cgi, accessed on 15 July 2026) to identify the bacterial strains [11].

### 2.3. Determination of Purine Clearance

Adenine, guanine, hypoxanthine, and xanthine (Sigma-Aldrich, Inc., St. Louis, MO, USA) were individually added to MRS defined medium to a final concentration of 40 mg per liter. Medium without added purines was used as a blank control. The activated bacterial suspension was inoculated into the purine containing medium at an inoculation level of 10% by weight and incubated under anaerobic conditions at 37 °C for 20 h. The fermentation broth was collected and centrifuged at 5000× *g* for 10 min at 4 °C. The supernatant was mixed with 10% perchloric acid at a volume ratio of nine to one, vortexed, and allowed to stand for 10 min, followed by centrifugation at 12,000× *g* for 10 min. The supernatant was filtered through a 0.22 μm membrane, and the residual concentrations of purines were determined by high performance liquid chromatography. The chromatographic analysis was performed using a Waters e2695 system (Waters Corporation, Milford, MA, USA) equipped with a T3 column (Waters Corporation) of 250 mm × 4.6 mm and particle size of 5 μm. The mobile phase consisted of 50 mmol per liter KH_2_PO_4_ at pH 4.0 and methanol at a volume ratio of 95 to 5. The separation was carried out under isocratic conditions with a flow rate of 1.0 mL per minute, a column temperature of 32 °C, a detection wavelength of 254 nm, and an injection volume of 10 μL. The concentrations of purines were calculated based on standard curves, and the clearance ratio was calculated as follows [12,13]:

(1) $$Clearance\;ratio\;(100\%)\;=\;\frac{C_{0}\;-\;C_{t}}{C_{0}}\;\times \;100\%$$

where C_0_ represents the purine concentration in the blank control group, expressed in milligrams per liter, and C_t_ represents the purine concentration in the sample group, also expressed in milligrams per liter. Five replicates were performed for each strain.

### 2.4. Determination of Antioxidant Activity of the Strain

#### 2.4.1. Determination of DPPH Radical Scavenging Activity

The activated strain was inoculated into MRS broth (Qingdao Hope Bio Technology Co., Ltd.) and incubated at 37 °C for 18 h. Five replicates were performed for each strain. The culture was centrifuged at 8000× *g* for 10 min at 4 °C to collect the cells, which were then washed twice with sterile phosphate buffered saline at pH 7.2. The cells were resuspended and adjusted to a concentration of 10^9^ CFU per milliliter. An aliquot of 1.0 mL of the bacterial suspension was mixed with 2.0 mL of 0.05 mmol per liter DPPH solution in ethanol and vortexed thoroughly. The mixture was incubated in the dark at room temperature for 30 min, followed by centrifugation at 8000× *g* for 10 min. The absorbance of the supernatant was measured at 517 nm. Deionized water was used instead of the bacterial suspension as the control group, and absolute ethanol was used instead of the DPPH solution as the blank group. The DPPH radical scavenging activity was calculated according to the following formula [14,15]:

(2) $$Clearance\;ratio\;(\%)\;=\;\left(1\;-\;\frac{A_{S}\;-\;A_{b}}{A_{c}}\right)\;\times \;100\%$$

where A_s_ represents the absorbance of the sample group, A_b_ represents the absorbance of the blank group, and A_c_ represents the absorbance of the control group. Five replicates were performed for each strain.

#### 2.4.2. Determination of Hydroxyl Radical Scavenging Activity

The reaction system consisted of 1.0 mL of 0.1% o-phenanthroline solution, 1.0 mL of PBS at pH 7.4, 1.0 mL of 2.5 mmol per liter FeSO_4_, 1.0 mL of 20 mmol per liter H_2_O_2_, and 0.5 mL of bacterial suspension at 10^9^ CFU per milliliter. After thorough mixing, the mixture was incubated in a water bath at 37 °C for 1.5 h, and the absorbance was measured at 536 nm. A system without bacterial suspension and H_2_O_2_ was used as the blank control, while a system without bacterial suspension was used as the model control. Ascorbic acid (Vc, 0.1 mg/mL) (Sinopharm Chemical Reagent Co., Ltd., Shanghai, China) was used as a positive control [16]. The hydroxyl radical scavenging activity was calculated according to the following equation [17,18]:

(3) $$Clearance\;ratio\;(100\%)\;=\;\left(1\;-\;\frac{A_{1}\;-\;A_{2}}{A_{1}\;-\;A_{3}}\right)\;\times \;100\%$$

where A_1_ represents the absorbance of the model control, A_2_ represents the absorbance of the sample group, and A_3_ represents the absorbance of the blank control. Five replicates were performed for each strain.

### 2.5. Determination of Biological Characteristics of the Strain

#### 2.5.1. Acid Tolerance

The activated bacterial suspension was centrifuged at 10,000× *g* for 10 min at 4 °C to collect the cells, which were then washed twice with PBS at pH 7.2 and resuspended in PBS adjusted to pH 3.0. The cell density was adjusted to 10^8^ CFU per milliliter. After incubation at 37 °C for 0 h and 3 h, samples were collected, serially diluted, and spread onto MRS agar plates (Qingdao Hope Bio Technology Co., Ltd.). The plates were incubated at 37 °C for 48 h, and viable colonies were counted. The survival rate was calculated as follows [19]:

(4) $$Survival\;rate\;(100\%)\;=\;\left(N_{t}/N_{0}\right)\;\times \;100\%$$

where N_t_ represents the viable cell count after treatment in CFU per milliliter, and N_0_ represents the initial viable cell count in CFU per milliliter. Five replicates were performed for each strain.

#### 2.5.2. Bile Salt Tolerance

The concentration of bile salts in the porcine digestive tract generally ranges from 0.03% to 0.3%. Therefore, PBS at pH 8.0 containing 0.3% porcine bile salts was prepared and sterilized by filtration through a 0.22 μm membrane. The washed bacterial cells were resuspended in the prepared bile salt solution and adjusted to a concentration of 10^8^ CFU per milliliter. After incubation at 37 °C for 0 h and 3 h, samples were collected for viable cell counting using the same method described above, and the survival rate was calculated as described in Section 2.5.1 [20]. Five replicates were performed for each strain.

#### 2.5.3. Tolerance to Gastrointestinal Digestive Enzymes

Pepsin (Sigma-Aldrich, Inc., St. Louis, MO, USA) was dissolved in sterile PBS at a concentration of 0.2 mol per liter and pH 3.0 to a final concentration of 3 g per liter, and sterilized by filtration through a 0.22 μm membrane. Trypsin (Sigma-Aldrich, Inc.) was dissolved in sterile PBS at a concentration of 0.2 mol per liter and pH 8.0 to a final concentration of 1 g per liter and sterilized in the same manner. All solutions were freshly prepared before use [21].

The bacterial suspension at 10^8^ CFU per milliliter was mixed with the prepared pepsin and trypsin solutions at a volume ratio of one to nine and incubated at 37 °C for 3 h. Subsequently, 1 mL of the culture was transferred into 9 mL of trypsin solution and incubated at 37 °C for an additional 8 h. Samples were collected after pepsin treatment at 0 h and 3 h, and after trypsin treatment at 2 h, 4 h, 6 h, and 8 h. Viable cell counts were determined, and survival rates were calculated as described above. Five replicates were performed for each strain.

### 2.6. Experimental Design and Animal Management

The experimental protocol used in the present study was approved by the Animal Care and Use Committee of the Institute of Subtropical Agriculture, Chinese Academy of Sciences (IACUC # 202431, Changsha, China).

A total of fourteen male diet-induced obese mice aged nine weeks were obtained from Beijing Vital River Laboratory Animal Technology Co., Ltd. (Beijing, China). The mice had been fed a high-fat diet for seven weeks before the experiment. The cage measured 43 cm × 17.9 cm × 17 cm, with 2–3 mice per cage. In addition, age-matched male C57BL/6J mice fed a standard diet were used as the normal control group, designated as group A, with seven animals. The diet-induced obese mice were randomly divided into two groups. Group B, with seven mice, continued to receive a high-fat diet and were administered saline by gavage. Group Y, with seven mice, continued to receive a high-fat diet and was administered a suspension of *P. acidilactici* (Pedio6-1). Mice in group Y were gavaged with 0.2 mL of bacterial suspension at a concentration of 1 × 10^9^ CFU per milliliter every other day, while mice in groups A and B received the same volume of saline. The experimental period lasted eight weeks. All mice were housed under controlled conditions at 22 to 25 °C with a 12 h light and dark cycle and had free access to food and water. Group A was fed a standard maintenance diet (Synergy Biotechnology Co., Ltd., Beijing, China), while groups B and Y were fed a high fat diet (Research Diets, Inc., New Brunswick, NJ, USA), formulation D12492. Detailed compositions of the diets are presented in Tables S1 and S2.

### 2.7. Sample Collection

At the end of the experiment, mice were euthanized by cervical dislocation. The body weight of each mouse was recorded. The whiskers and hair around the orbital area were removed, and blood samples were collected by enucleation using forceps. The blood was allowed to stand at room temperature for 2 h, followed by storage at 4 °C for 4 h, and then centrifuged at 4000× *g* for 10 min to separate the serum. The serum was aliquoted and stored at −20 °C for further analysis. The abdominal cavity was then opened, and the spleen, liver, kidneys, heart, inguinal white adipose tissue (iWAT), and epididymal white adipose tissue (eWAT) were promptly removed and weighed. The organ index was calculated as (organ weight/body weight) × 100% and expressed as a percentage. The colon was isolated, and colonic contents were collected, immediately frozen in liquid nitrogen, and stored at −80 °C for subsequent microbial diversity analysis.

### 2.8. Determination of Purine Content in Mouse Liver

Approximately 0.2 g of liver tissue was weighed and transferred into a 10 mL stoppered graduated centrifuge tube. Three milliliters of 10% perchloric acid was added, and the mixture was hydrolyzed in a water bath at approximately 99 °C for 60 min with intermittent shaking to ensure uniform dispersion. After cooling, the pH was adjusted to 7.0 ± 0.1 using 1 mol/L KOH, and the volume was brought to 10 mL and mixed thoroughly. The mixture was centrifuged at 6000 r per minute for 10 min, and the supernatant was collected [22]. The supernatant was filtered through a 0.22 μm syringe filter prior to analysis. According to the method described in Section 2.3, HPLC was used to determine the concentrations of xanthine, adenine, guanine, hypoxanthine, and their related metabolites including creatinine, adenosine, and guanosine in liver samples.

### 2.9. Real Time-Quantitative PCR

The mRNA expression levels of inflammation-related genes, including tumor necrosis factor-α (TNF-α), interleukin (IL)-1β, IL-6, IL-10, Toll-like receptor (TLR) 4, and TLR8, were measured in the liver using real-time quantitative PCR. The primer sequences employed in this study are provided in Supplementary Table S4. Total RNA was isolated with TRIzol reagent (Invitrogen, Thermo Fisher Scientific Inc., Carlsbad, CA, USA), and its concentration was assessed using a NanoDrop 2000 spectrophotometer (Thermo Fisher Scientific Inc., Wilmington, DE, USA). Complementary DNA was synthesized using the PrimeScript^TM^ RT reagent kit with gDNA Eraser (Takara Bio Inc., Shiga, Japan). β-Actin served as the internal reference gene for normalizing target gene transcript levels. Quantitative PCR was carried out in a 10 μL reaction mixture using the SYBR Premix Ex Taq kit (Takara Bio Inc.). The thermal cycling protocol followed that described in a previous study [23].

### 2.10. Serum Metabolomics Analysis

Serum samples were subjected to untargeted metabolomics analysis using ultra high-performance liquid chromatography coupled with tandem mass spectrometry (Waters Corporation, Milford, MA, USA). Sample preparation, chromatographic separation, and mass spectrometry conditions were performed as previously described [24]. The raw data were processed using Progenesis QI software (Version 3.0, Waters Corporation, Milford, MA, USA) for peak detection, alignment, and normalization. Metabolites were identified by comparison with databases including HMDB (The Metabolomics Innovation Center, Edmonton, AB, Canada) and METLIN (Scripps Center for Metabolomics, The Scripps Research Institute, La Jolla, CA, USA). Multivariate statistical analysis was applied to screen differential metabolites, with variable importance in projection values greater than 1 and *p* < 0.05 from the *t* test used as selection criteria. Pathway enrichment analysis of differential metabolites was conducted based on the KEGG database [25].

### 2.11. Statistical Analysis

All data are expressed as mean ± standard error of the mean (SEM). Statistical analyses were performed using SPSS software SPSS software (version 25.0, IBM Corp., Armonk, NY, USA). For comparisons between two groups, unpaired two-tailed Student’s *t*-tests were applied. Normality of residuals was assessed using the Shapiro–Wilk test for each group across all comparisons, with *p* > 0.05 indicating no significant deviation from a normal distribution. For multi-group comparisons, one-way analysis of variance (ANOVA) was employed, followed by Tukey’s honest significant difference (HSD) post hoc test for multiple pairwise comparisons. Homogeneity of variances was evaluated using Levene’s test. Statistical significance was defined as *p* < 0.05, while 0.05 ≤ *p* < 0.10 was considered a trend toward significance. In all figures, distinct lowercase letters above the bars denote statistically significant differences among groups (*p* < 0.05).

## 3. Results

### 3.1. Identification and Growth Curves of the Isolates

**Figure 1:**
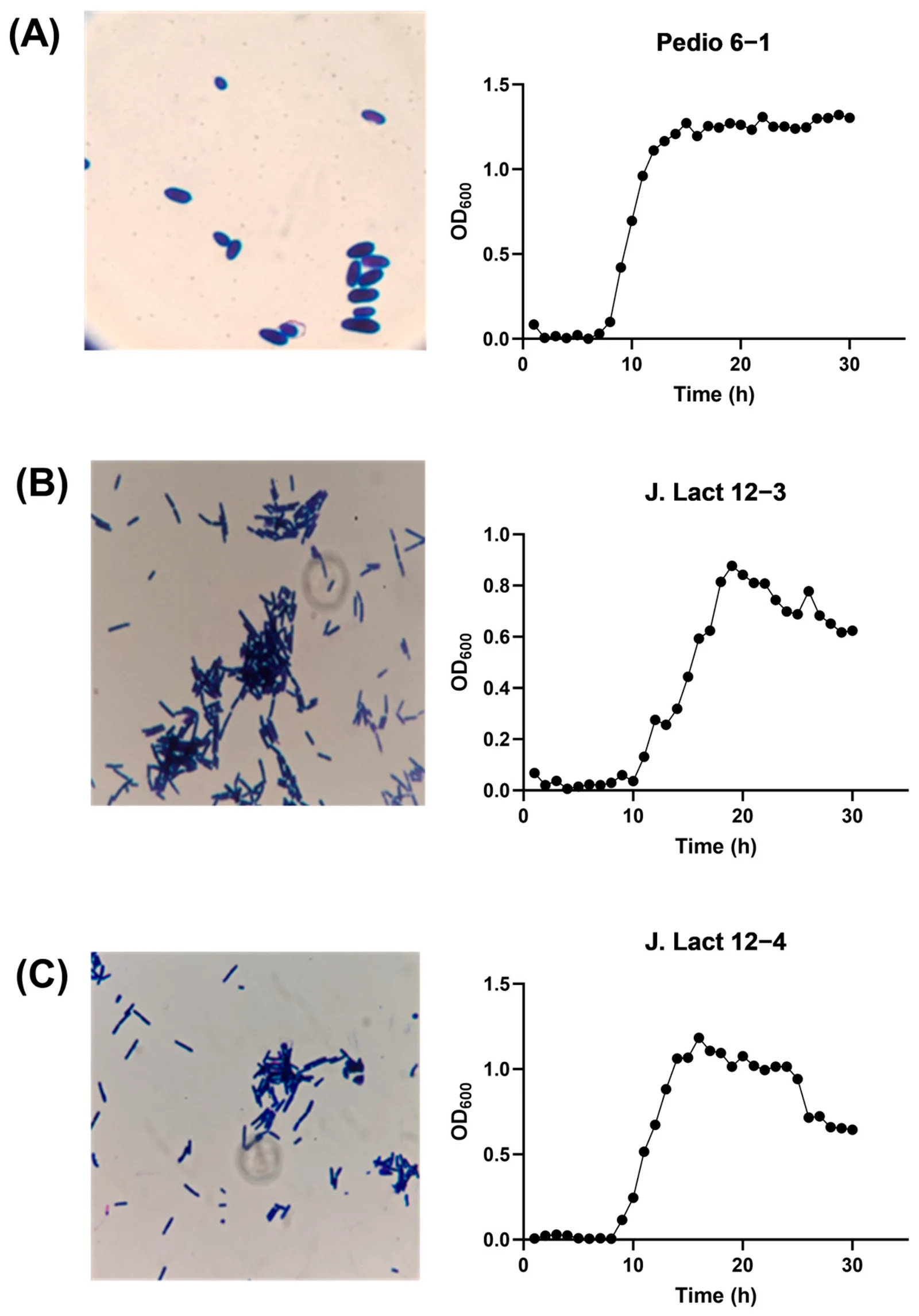
Gram staining morphology (magnification 100×) and growth curve of (**A**) Pedio6-1, (**B**) J.Lact 12-3, and (**C**) J.Lact 12-4. Data are presented as mean ± SEM (n = 3).

Based on microscopic observation and sequence alignment analysis, a total of three lactic acid bacterial strains were obtained. Among them, one strain was identified as *Pediococcus acidilactici* designated Pedio6-1, while the other two strains were identified as *Lactobacillus johnsonii* designated J.Lact 12-3 and J.Lact 12-4. The assembled sequences are presented in Table S3. The microscopic characteristics and growth curves of the three lactic acid bacterial strains are shown in Figure 1.

### 3.2. In Vitro Purine Clearance Capacity of the Isolates

**Figure 2:**
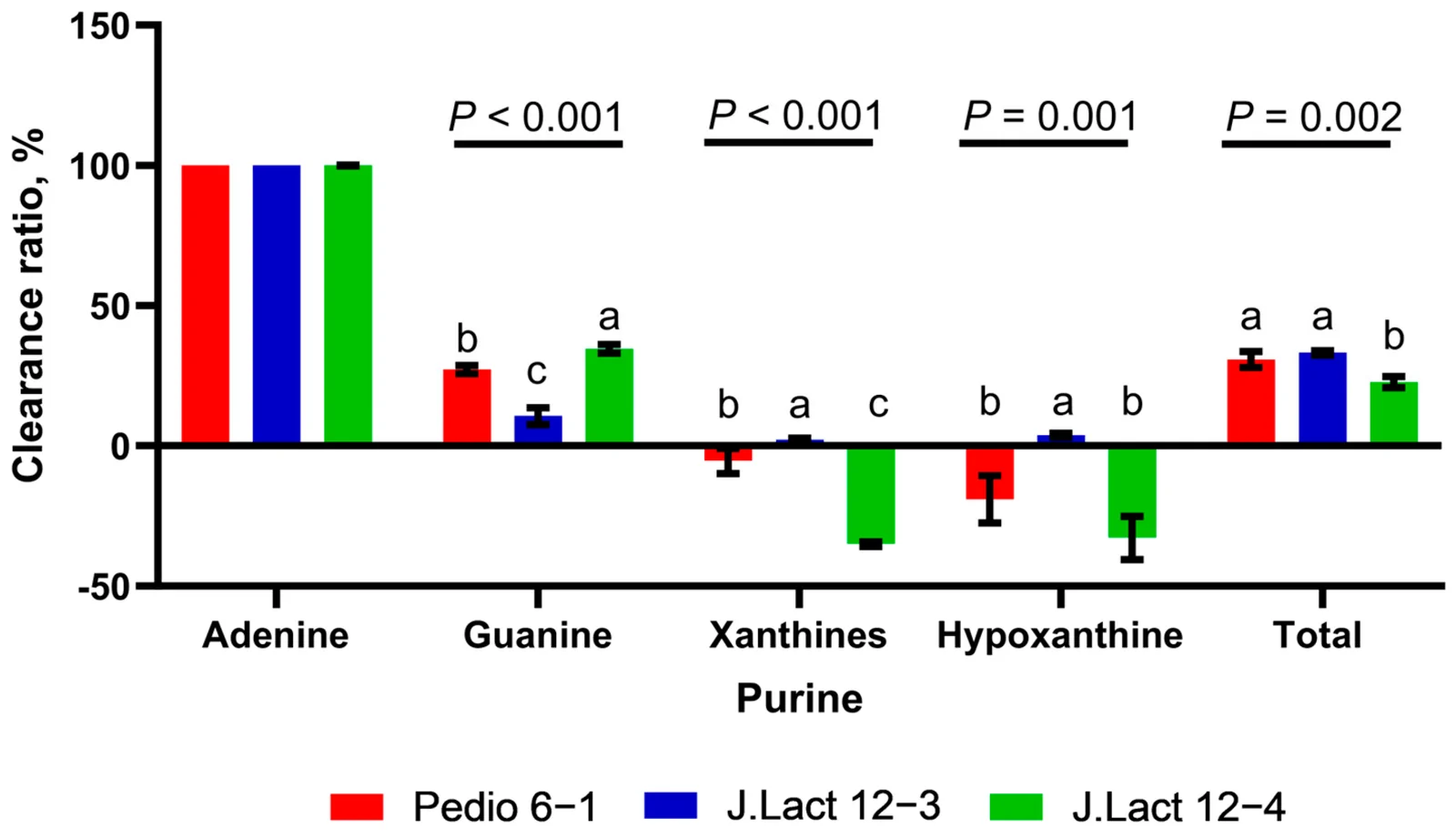
Purine clearance rates of the three isolated strains. Data are presented as mean ± SEM (n = 5). Different lowercase letters above the bars indicate statistically significant differences among groups (*p* < 0.05).

The in vitro purine clearance capacities of the three isolated strains were evaluated, and the results are presented in Figure 2. All three strains exhibited nearly complete removal of adenine, with clearance rates approaching 100%. The guanine clearance rate of J.Lact 12-4 was significantly higher than that of Pedio6-1 and J.Lact 12-3 (*p* < 0.05). In contrast, J.Lact 12-3 showed significantly higher clearance rates for xanthine and hypoxanthine compared with the other strains (*p* < 0.05). In terms of total purine clearance, Pedio6-1 and J.Lact 12-3 exhibited significantly higher values than J.Lact 12-4 (*p* < 0.05).

### 3.3. Antioxidant Activity of the Isolates

**Figure 3:**
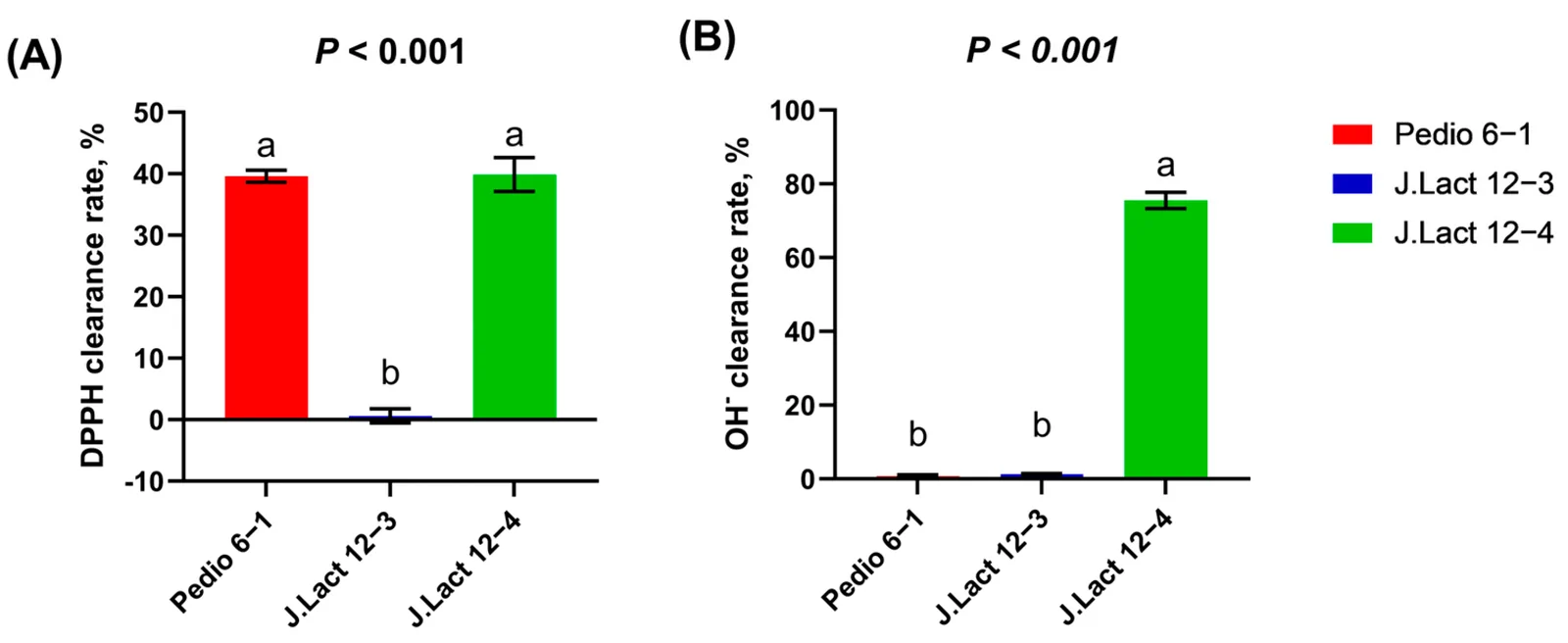
Antioxidative properties of the three isolated strains. (**A**) DPPH radical scavenging activity. (**B**) Hydroxyl radical (OH^−^) scavenging activity. Data are presented as mean ± SEM (n = 5). Different lowercase letters above the bars indicate statistically significant differences among groups (*p* < 0.05).

The positive control (ascorbic acid, 0.1 mg/mL) exhibited a hydroxyl radical scavenging activity of 86.3% ± 1.5% (DPPH) and 90.7% ± 3.6% (OH^−^), confirming that the assay system was functioning properly and the results obtained for the tested strains were reliable. The in vitro hydroxyl radical scavenging activity and DPPH radical scavenging activity of the three strains were evaluated, and the results are shown in Figure 3. J.Lact 12-4 exhibited a hydroxyl radical scavenging rate of approximately 80%, which was significantly higher than that of J.Lact 12-3 and Pedio6-1 (*p* < 0.05). Both Pedio6-1 and J.Lact 12-4 showed DPPH radical scavenging activities of approximately 40%, and these values were significantly higher than that of J.Lact 12-3 (*p* < 0.05).

### 3.4. Gastrointestinal Tolerance of the Isolates

**Figure 4:**
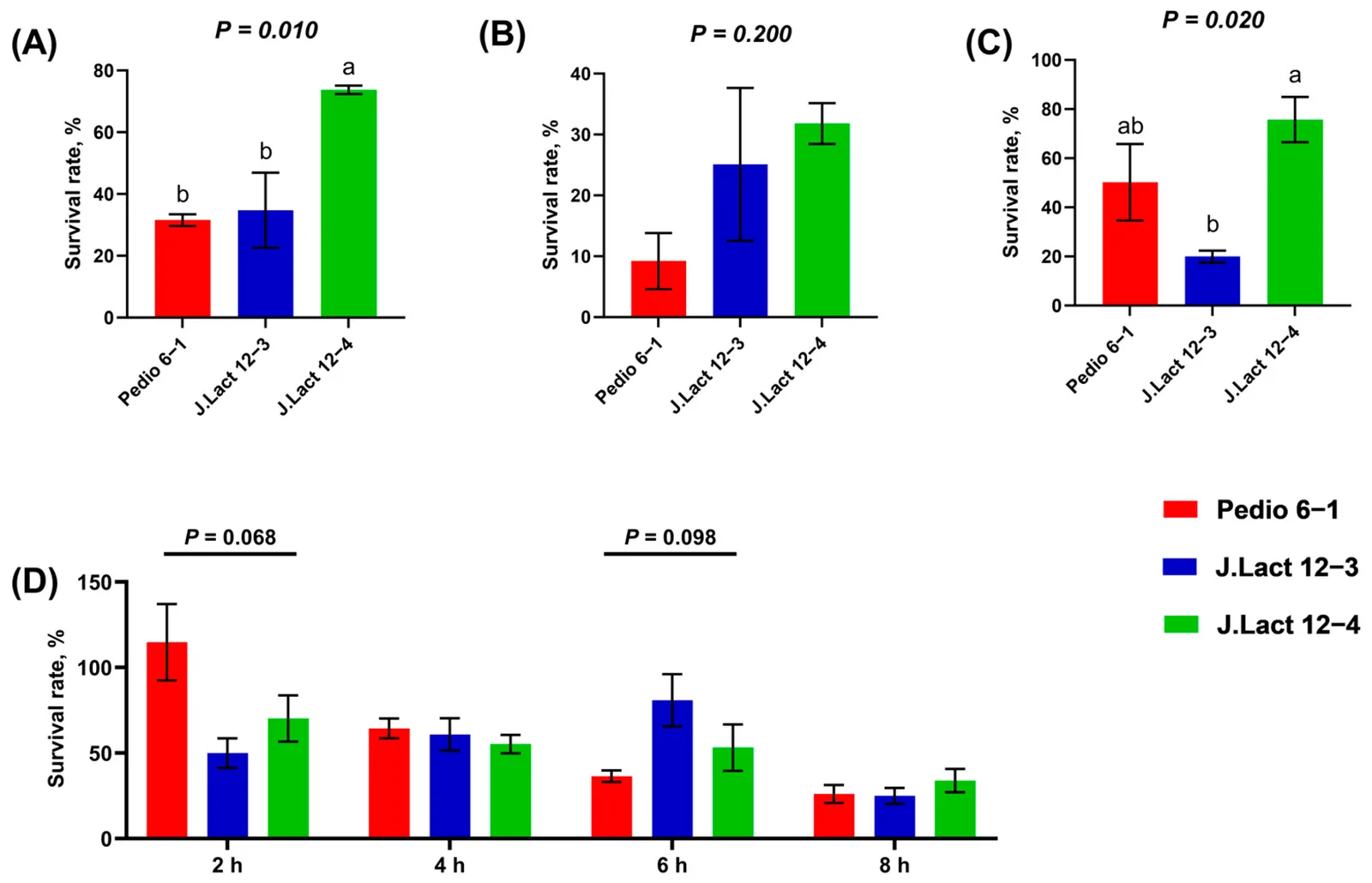
Gastrointestinal tract tolerance of the three isolated strains. (**A**) Acid tolerance (pH 3.0, 3 h). (**B**) Bile salt tolerance (0.3%, 3 h). (**C**) Pepsin tolerance (3 g/L, pH 3.0, 3 h). (**D**) Trypsin tolerance (1 g/L, pH 8.0, 8 h). Data are presented as mean ± SEM (n = 5). Different lowercase letters above the bars indicate statistically significant differences among groups (*p* < 0.05).

The gastrointestinal tolerance of the three strains was evaluated, and the results are shown in Figure 4. After incubation at pH 3 for 3 h, J.Lact 12-4 exhibited the highest survival rate, exceeding 70%, which was significantly higher than that of J.Lact 12-3 and Pedio6-1 (*p* < 0.05). After exposure to 0.3% bile salts for 3 h, J.Lact 12-4 also showed the highest survival rate at approximately 35%, while Pedio6-1 showed the lowest survival. Following pepsin treatment for 3 h, the survival rate of J.Lact 12-4 was approximately 80%, which was significantly higher than that of J.Lact 12-3 (*p* < 0.05). In the trypsin tolerance assay, Pedio6-1 showed a higher survival rate after 2 h of incubation (*p* < 0.05), whereas after 8 h of incubation, the survival rates of all strains were approximately 30%, with J.Lact 12-4 showing the highest survival rate of 33.87% (*p* < 0.05).

### 3.5. Growth Curve and Organ Indices of Mice

**Figure 5:**
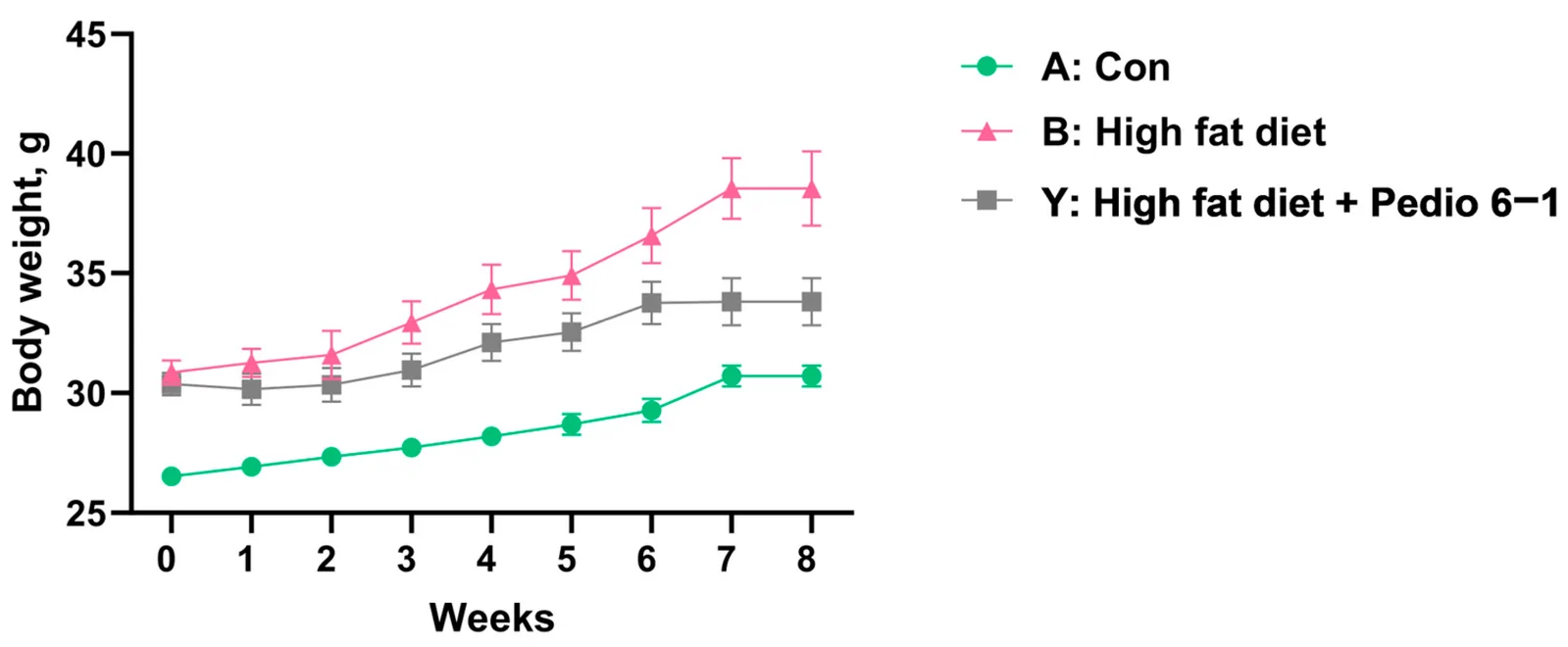
Growth curve of mice over the 8-week treatment period. Body weight was recorded weekly. Data are presented as mean ± SEM (n = 7). A: normal control group; B: high-fat diet group; Y: high-fat diet + Pedio6-1 group.

Given that the in vitro assays showed that *Pedio6-1* exhibited the highest purine clearance efficiency, together with antioxidant activity and tolerance to trypsin and bile salts, this strain was selected for further in vivo experiments. After 8 weeks of treatment, the results shown in Figure 5 and Table 1 indicated that compared with group B, mice in group Y treated with Pedio6-1 exhibited significantly reduced final body weight and liver index (*p* < 0.05).

### 3.6. Purine Content in Mouse Liver

The purine contents in mouse liver are presented in Table 2. The diet-induced obese mice (group B) showed significantly higher levels of xanthine, guanine, and total purines in the liver compared with normal control mice (group A) at *p* < 0.05. After the administration of Pedio6-1, diet-induced obese mice (group Y) exhibited a significant reduction in hepatic guanine content (*p* < 0.05). In addition, hypoxanthine levels were significantly decreased (*p* < 0.05), while the total purine content showed a decreasing trend.

### 3.7. Relative mRNA Expressions of Pro-Inflammatory Cytokines and Toll-like Receptor in Liver

**Figure 6:**
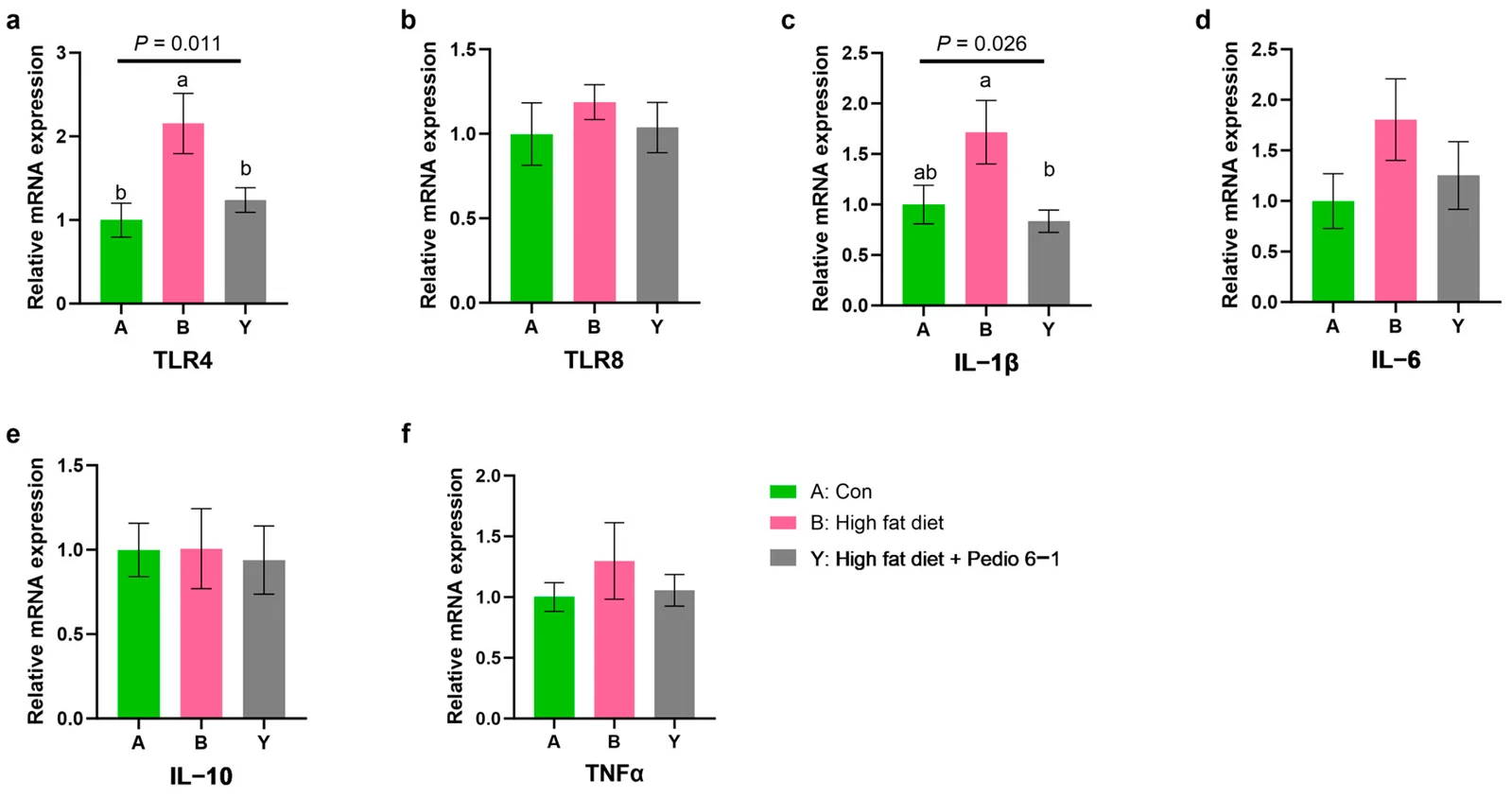
Effects of Pedio6-1 on the relative mRNA expression of inflammatory cytokines and Toll-like receptors in the liver. Data are presented as mean ± SEM (n = 7). Different lowercase letters above the bars indicate statistically significant differences among groups (*p* < 0.05). A: normal control group; B: high-fat diet group; Y: high-fat diet + Pedio6-1 group. (**a**) TLR4; (**b**) TLR8; (**c**) IL-1β; (**d**) IL-6; (**e**) IL-10; (**f**) TNFα.

As shown in Figure 6, compared with healthy mice (group A), a high-fat diet (group B) led to a significant upregulation of the mRNA expression of TLR4 in the liver (*p* < 0.05). Compared with group B, Pedio6-1 (group Y) significantly downregulated the mRNA expression of IL-1β and TLR4 (*p* < 0.05), and the data were indistinguishable from those of healthy mice (group A).

### 3.8. Serum Metabolome Profile

**Figure 7:**
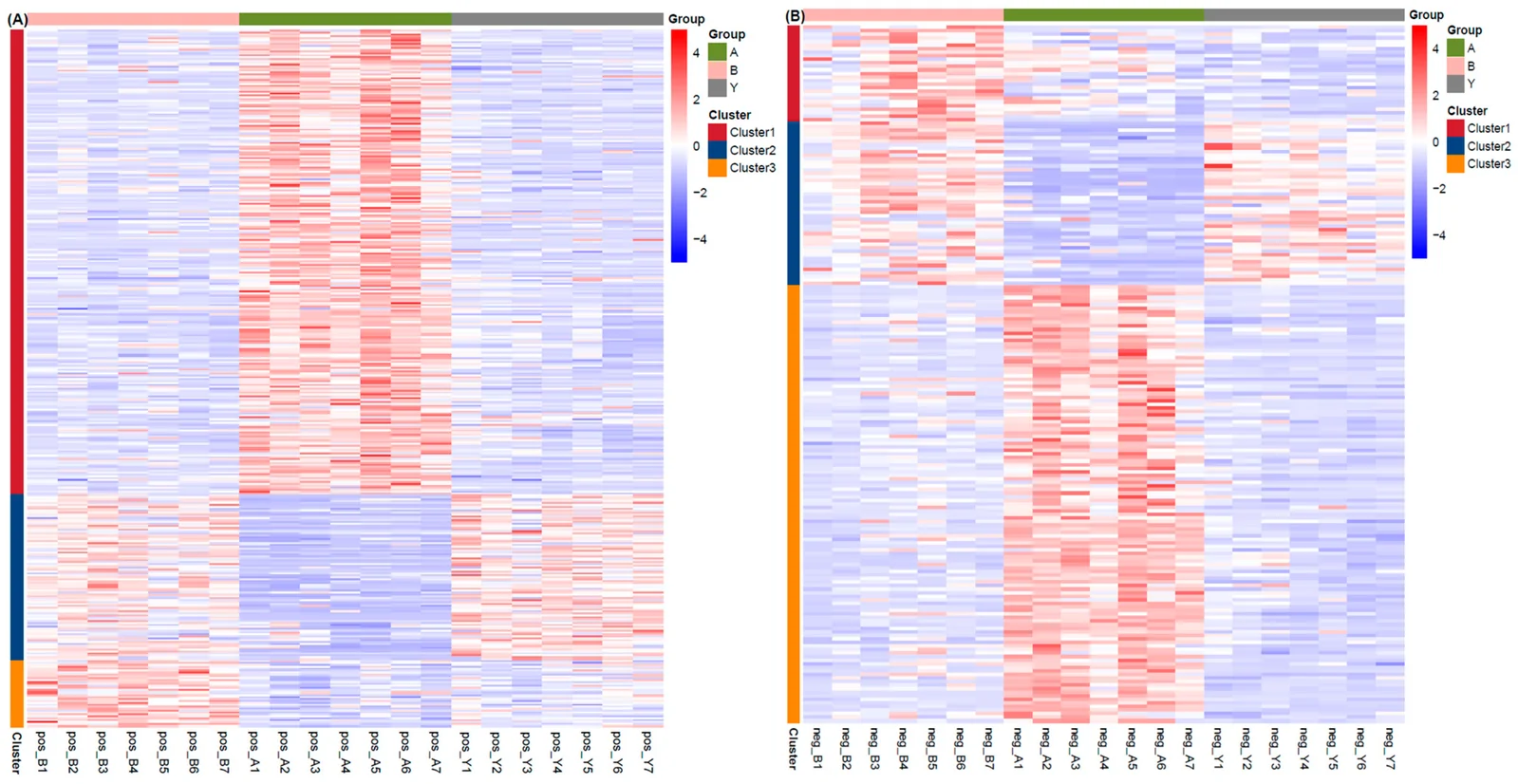
Differential metabolite heatmaps in positive and negative ion modes. (**A**) Positive ion mode. (**B**) Negative ion mode. Data are presented as n = 7 per group. A: normal control group; B: high-fat diet group; Y: high-fat diet + Pedio6-1 group. Red indicates upregulated metabolites; blue indicates downregulated metabolites.

**Figure 8:**
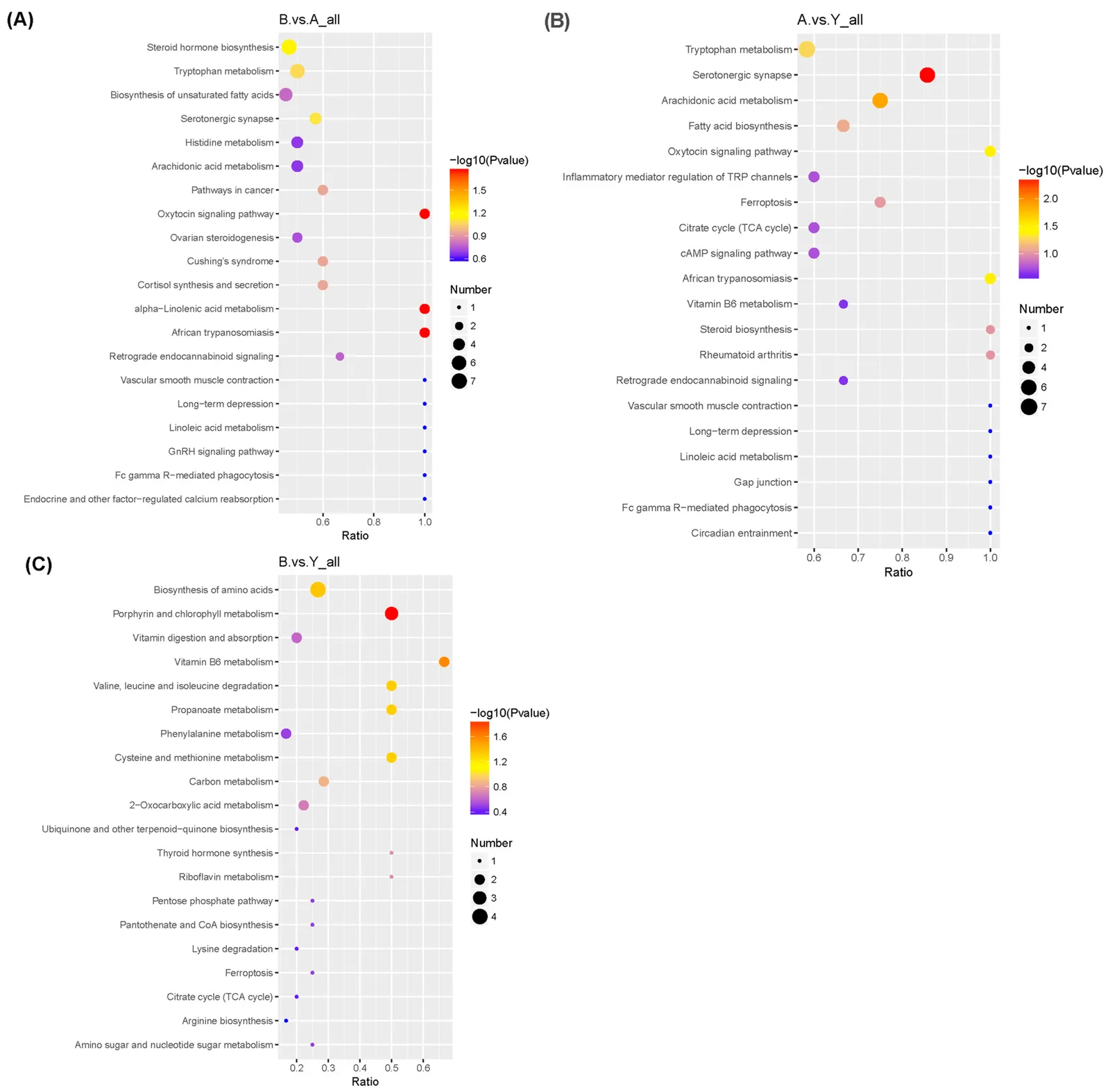
KEGG pathway enrichment analysis of differential metabolites between groups: (**A**) group B vs. group A; (**B**) group A vs. group Y; (**C**) group B vs. group Y. The size of the circles represents the number of metabolites enriched in each pathway, and the color gradient represents the *p*-value. Data are presented as n = 7 per group. A: normal control group; B: high-fat diet group; Y: high-fat diet + Pedio6-1 group.

Serum samples were collected from mice for untargeted metabolomics analysis. The results showed that there were 436 differential metabolites in A vs. B, 105 differential metabolites in A vs. Y, and 449 differential metabolites in B vs. Y. Heatmap analysis (Figure 7) indicated that the serum metabolic profile of group B was clearly distinct from that of the other two groups, whereas supplementation with Pedio6-1 shifted the metabolic profile of group Y closer to that of group A. KEGG pathway enrichment analysis revealed that the differential pathways between diet-induced obese mice (group B) and Pedio6-1 treated mice (group Y) were mainly enriched in porphyrin metabolism as well as amino acid biosynthesis and metabolism (Figure 8). Further *t* test analysis of key metabolites in these pathways (Table 3) showed that porphobilinogen, bilirubin, biliverdin, D erythrose 4 phosphate, L cystathionine, citrate, glutathione, and methylmalonic acid were significantly increased (*p* < 0.05), while 4 pyridoxic acid showed an increasing trend (0.05 < *p* < 0.1). In contrast, N acetylornithine and methylmalonate ester were significantly decreased (*p* < 0.05).

## 4. Discussion

In this study, a lactic acid bacterial strain with potential purine-degrading ability, *Pediococcus acidilactici* (Pedio6-1), was isolated from the colonic contents of Daweizi pigs fed a low-protein diet without soybean meal. In vitro functional characterization showed that Pedio6-1 exhibited an adenine clearance rate close to 100% and a total purine clearance rate of 30.73%, which was superior to that of certain *Lactobacillus johnsonii* strains. These findings indicate that Pedio6-1 possesses strong purine degradation capacity and may reduce host purine burden by directly degrading purine compounds in the intestinal lumen and thereby limiting their absorption. Previous studies have also reported that lactic acid bacteria such as *Lactobacillus gasseri* PA-3 and *Lactobacillus brevis* DM9218 exhibit purine degrading activity, which is likely associated with their intrinsic purine metabolism related enzymes, including purine nucleoside phosphorylase and xanthine oxidase [26,27]. In addition, Pedio6-1 showed strong antioxidant activity, as evidenced by its DPPH and hydroxyl radical scavenging abilities, suggesting its potential to alleviate oxidative stress induced by high purine diets. The strain also demonstrated favorable gastrointestinal tolerance, with a survival rate exceeding 70% at pH 3.0, approximately 35% in 0.3% bile salts, and relatively high survival after exposure to pepsin and trypsin. These characteristics indicate that Pedio6-1 is capable of surviving gastrointestinal barriers and may colonize the intestinal tract effectively to exert probiotic functions. Gastrointestinal tolerance is a key prerequisite for probiotic functionality, and acid and bile resistance are widely recognized as essential screening criteria for probiotic candidates.

Based on its superior in vitro performance, including high purine clearance, antioxidant capacity, and resistance to digestive stress, *Pedio6-1* was selected for in vivo evaluation. Currently, mouse models used to study purine metabolism disorders can be classified into two types: gene-edited models and chemically or diet-induced models. Among these, diet-induced models primarily involve the oral administration of compounds such as potassium oxonate, xanthine, hypoxanthine, and nicotinic acid [28]. Studies have shown that obese male mice commonly exhibit purine metabolism disorder and elevated uric acid levels, with males exhibiting more severe symptoms than females [29]. It is reported to be associated with insulin resistance, which promotes uric acid reabsorption [30]. In addition, adipose tissue-derived inflammatory cytokines such as TNF-α and IL-6 are elevated in obesity and may interfere with enzymes involved in purine metabolism, further increasing uric acid production [31]. Therefore, we used high-fat diet-induced obese male mice as the animal model, not only to evaluate the effect of Pedio6-1 on regulating purine metabolism, but also to assess its impact on obesity [32].

The liver is the primary organ for purine metabolism, playing central roles in purine nucleotide synthesis, degradation, and salvage pathways [33,34]. In this study, Pedio6-1 administration significantly reduced the final body weight and liver index in diet-induced obese mice. Hepatic levels of xanthine, guanine, and total purines were significantly higher in obese mice compared with normal controls, indicating that a high fat diet induced purine metabolic disorder. Treatment with Pedio6-1 significantly reduced the hepatic guanine levels and showed an increasing trend in hypoxanthine, while total purine levels tended to decrease. Similar findings have been reported for other lactic acid bacteria. For example, *Lactobacillus paracasei* X11 can completely degrade nucleotides and nucleosides within 30 min in vitro, with degradation rates of 83.25% for purine and 80.42% for oxopurine compounds. Moreover, lactic acid bacteria may reduce uric acid production by inhibiting xanthine oxidase activity through short chain fatty acid mediated mechanisms. Strains such as *Lactobacillus rhamnosus* R31, *Lactobacillus rhamnosus* R28 1, and *Lactobacillus reuteri* L20M3 have been shown to significantly reduce the serum and urinary uric acid levels as well as xanthine oxidase activity in serum and liver tissues in hyperuricemic mice [35]. Serum metabolomics analysis revealed that the metabolic profile of Pedio6-1 treated mice was more similar to that of normal mice. KEGG pathway enrichment analysis indicated that the differential pathways between obese mice and Pedio6-1 treated mice were mainly associated with porphyrin metabolism and amino acid metabolism. These two pathways play essential roles in cellular function, energy metabolism, and biosynthesis.

Further statistical analysis of key metabolites in these pathways showed that Pedio6-1 treatment significantly increased the serum levels of porphobilinogen, bilirubin, biliverdin, 4-pyridoxic acid, L-cystathionine, and citrate. Porphobilinogen is a pyrrole compound involved in porphyrin metabolism and serves as an intermediate in heme biosynthesis, and its elevation is associated with disorders of hepatic porphyrin and heme metabolism [36]. Bilirubin, a degradation product of heme, is typically reduced in obesity. Obesity is associated with increased hepatic glucuronidation activity that enhances bilirubin clearance, and serum bilirubin levels are negatively correlated with visceral obesity and hypertriglyceridemia [37]. Biliverdin reductase converts biliverdin into bilirubin, contributing to the reduction in oxidative stress and obesity-related metabolic disorders [38].

The metabolomic data further revealed that Pedio6-1 treatment coordinately modulated multiple interconnected pathways, including vitamin B6 metabolism, transsulfuration, TCA cycle, and pentose phosphate pathway. Specifically, Pedio6-1 significantly elevated the serum levels of 4-pyridoxic acid, D-erythrose 4-phosphate, L-cystathionine, citrate, and glutathione, while decreasing N-acetylornithine and methylmalonate ester in obese mice. The elevation of 4-pyridoxic acid and D-erythrose 4-phosphate—metabolites linked to vitamin B6 turnover and aromatic amino acid precursor supply—suggests a restoration of vitamin B6 homeostasis, which is often impaired in obesity [39,40,41,42,43]. More importantly, the significant upregulation of L-cystathionine and glutathione (2.0-fold increase) indicates enhanced flux through the transsulfuration pathway, which is critical for maintaining cellular redox balance. Given that glutathione depletion is a hallmark of high-fat diet-induced oxidative stress [44], this increase aligns consistently with the strain’s in vitro antioxidant activity (Figure 3) and the downregulation of hepatic IL-1β and TLR4 expression (Figure 6), supporting that Pedio6-1 alleviates obesity-associated inflammation partly through bolstering the glutathione antioxidant axis.

The concurrent elevation of citrate—a TCA cycle intermediate and key regulator of glycolysis and lipogenesis—suggests improved mitochondrial function and metabolic flexibility in Pedio6-1-treated mice [42]. Additionally, the decreased N-acetylornithine and methylmalonate ester point to improved nitrogen disposal and altered branched-chain amino acid/propionate metabolism, respectively, reflecting broader metabolic remodeling.

Collectively, these coordinated metabolomic changes demonstrate that Pedio6-1 not only degrades purines, but also reverses multiple obesity-related metabolic disturbances, including oxidative stress, vitamin B6 dysregulation, and TCA cycle perturbation. These findings provide a mechanistic framework for the observed reductions in body weight, liver index, and hepatic guanine content, and further reinforce the probiotic potential of Pedio6-1 in managing obesity-associated purine metabolic disorders.

## 5. Conclusions

In this study, a strain of *Pediococcus acidilactici* Pedio6-1 was isolated and identified from the intestinal contents of Daweizi pigs. This strain exhibited efficient purine clearance capacity in vitro, with a total purine clearance rate of 30.73% and an adenine clearance rate of 100%, and showed strong tolerance to acidic conditions, bile salts, and gastrointestinal digestive enzymes. In a diet-induced obese mouse model, Pedio6-1 intervention significantly reduced the final body weight and liver index, decreased the hepatic guanine content, and shifted the serum metabolic profile toward that of normal mice. KEGG pathway enrichment analysis indicated that Pedio6-1 primarily regulated porphyrin metabolism and the amino acid biosynthesis and metabolism pathways, accompanied by significant increases in metabolites with antioxidant functions, including bilirubin, biliverdin, and glutathione. Collectively, these findings suggest that Pedio6-1 has the potential to modulate purine metabolism and ameliorate obesity-related metabolic disorders, and therefore represents a promising candidate for further development as a functional probiotic.

## Figures and Tables

**Table 1:** Effects of Pedio6-1 on body weight and organ indices in DIO mice.

Groups	A	B	Y	*p*-Value
Final body weight	30.71 ± 1.1 ^b^	38.54 ± 4.10 ^a^	33.54 ± 2.59 ^b^	0.001
Liver index	3.46 ± 0.38 ^a^	3.37 ± 0.22 ^a^	2.96 ± 0.23 ^b^	0.010
Kidney index	1.14 ± 0.15 ^a^	0.88 ± 0.09 ^b^	0.98 ± 0.08 ^b^	0.001
Spleen index	0.24 ± 0.03 ^a^	0.17 ± 0.02 ^b^	0.20 ± 0.02 ^b^	<0.001
Heart index	0.55 ± 0.06 ^a^	0.42 ± 0.06 ^b^	0.47 ± 0.09 ^b^	0.007
iWAT index	0.83 ± 0.21 ^b^	4.08 ± 0.63 ^a^	3.93 ± 1.51 ^a^	<0.001
eWAT index	2.14 ± 0.73 ^b^	7.00 ± 0.78 ^a^	6.08 ± 1.20 ^a^	<0.001

Data are presented as mean ± SEM (n = 7). A: normal control group (standard diet); B: high-fat diet group (HFD + saline); Y: high-fat diet + Pedio6-1 group (HFD + Pedio6-1). Different lowercase letters within the same row indicate statistically significant differences among groups (*p* < 0.05). iWAT: inguinal white adipose tissue; eWAT: epididymal white adipose tissue.

**Table 2:** Effects of Pedio6-1 on hepatic purine content in DIO mice.

Purines (μg/g)	A	B	Y	*p*
Zanthine	220.73 ± 7.28 ^b^	507.11 ± 54.50 ^a^	458.40 ± 54.69 ^a^	0.001
Guanine	1415.01 ± 43.97 ^b^	1630.47 ± 44.26 ^a^	1443.37 ± 14.88 ^b^	0.002
Hypoxanthine	576.87 ± 16.34 ^b^	592.04 ± 23.86 ^b^	702.69 ± 24.41 ^a^	0.001
Adenine	93.23 ± 15.76	77.11 ± 4.65	69.26 ± 3.86	0.252
Total	2274.31 ± 57.155 ^b^	2806.73 ± 99.54 ^a^	2411.18 ± 158.91 ^ab^	0.005

Data are presented as mean ± SEM (n = 7). A: normal control group; B: high-fat diet group; Y: high-fat diet + Pedio6-1 group. Different lowercase letters within the same row indicate statistically significant differences among groups (*p* < 0.05).

**Table 3:** Key differential serum metabolites identified in mice.

Metabolites	B vs. A		Y vs. B		Y vs. A		Related Pathway
	Ratio	Change	Ratio	Change	Ratio	Change	
Jasmonic acid	0.651	↓	–	–	–	–	alpha-Linolenic acid metabolism
Traumatic acid	0.082	↓	–	–	–	–	alpha-Linolenic acid metabolism
13(S)-HOTrE	1.655	↑	–	–	–	–	alpha-Linolenic acid metabolism
Arachidonic acid	0.617	↓	–	–	1.607	↑	Oxytocin signaling pathway
Prostaglandin E2	2.068	↑	–	–	0.330	↓	Oxytocin signaling pathway
Prostaglandin H2	26.46	↑	–	–	0.030	↓	Oxytocin signaling pathway
L-Tryptophan	3.07	↑	–	–	0.1996	↓	Serotonergic synapse
Serotonin	–	–	–	–	0.504	↓	Serotonergic synapse
Thromboxane B2	–	–	–	–	0.521	↓	Serotonergic synapse
5-OxoETE	–	–	–	–	0.327	↓	Arachidonic acid metabolism
16(R)-HETE	–	–	–	–	0.637	↓	Arachidonic acid metabolism
Porphobilinogen	–	–	1.716	↑	–	–	Porphyrin and chlorophyll metabolism
Bilirubin	–	–	3.117	↑	–	–	Porphyrin and chlorophyll metabolism
Biliverdin	–	–	1.604	↑	–	–	Porphyrin and chlorophyll metabolism
4-Pyridoxic acid	–	–	1.618	↑	–	–	Vitamin B6 metabolism
D-Erythrose 4-phosphate	–	–	2.889	↑	–	–	Vitamin B6 metabolism
L-Cystathionine	–	–	1.770	↑	–	–	Biosynthesis of amino acids; cysteine and methionine metabolism
N-Acetylornithine	–	–	0.346	↓	–	–	Biosynthesis of amino acids
Citric acid	–	–	1.772	↑	–	–	Biosynthesis of amino acids
Glutathione	–	–	2.000	↑	–	–	Cysteine and methionine metabolism
Methylmalonic acid	–	–	2.278	↑	–	–	Valine, leucine and isoleucine metabolism; propanoate metabolism
Methylmalonate	–	–	0.573	↓	–	–	Valine, leucine and isoleucine metabolism; propanoate metabolism

Data are presented as n = 7 per group. A: normal control group; B: high-fat diet group; Y: high-fat diet + Pedio6-1 group. Ratio represents fold change; ↑ indicates upregulation; ↓ indicates downregulation; – indicates no significant change. Only metabolites with significant differences (*p* < 0.05) or trends (0.05 ≤ *p* < 0.10) are shown.

## Data Availability

The original contributions presented in this study are included in the article/Supplementary Materials. Further inquiries can be directed to the corresponding author.

## Supplementary Materials

The following supporting information can be downloaded at: https://www.mdpi.com/article/10.3390/microorganisms14081677/s1, Table S1: Nutrition level of basal diet; Table S2: Composition and Nutrition level of high-fat diet. Table S3: The 16S region gene sequences of *Pedio. 6-1*, *J.Lact 12-3*, and *J.Lact 12-4*. Table S4: Primers used for quantitative PCR.
