# Supplementary material for: In Vitro and In Vivo Evaluation of Pediococcus acidilactici Pedio6-1 for Purine Metabolism Modulation in Diet-Induced Obese Mice
Source: Microorganisms. 2026 Jul 30;14(8):1677. doi: 10.3390/microorganisms14081677 (PMC13515496; doi:10.3390/microorganisms14081677)
Supplement: Supplementary file 1 [file microorganisms-14-01677-s001.zip › microorganisms-4313540-supplementary.pdf]

Supplementary Information

In *vitro* and in *vivo* evaluation of *Pediococcus acidilactici* Pedio6-1 for purine metabolism modulation in diet-induced obese mice

Haohua Fu[1], Hengjia Ni[2,3,*], Jianhui Wang[1], Tuo Leng[1], Shusong Wu[2], Pan Huang[3], Jianjun Li[3], Cimin Long[3], Yulong Yin[2,3,*]

[1] School of Food Science and Bioengineering, Changsha University of Science and Technology, Changsha 410114, China; rayaiyz@163.com (H.F.); wangjh0909@163.com (J.W.); lengtuo95@163.com (T.L.)

[2] College of Animal Science and Technology, Hunan Agriculture University, Changsha 410125, China; wush688@hunau.edu.cn

[3] Key Laboratory of Agro-Ecological Processes in Subtropical Region, Hunan Provincial Key Laboratory of Animal Nutritional Physiology and Metabolic Process, Hunan Research Center of Livestock and Poultry Sciences, South Central Experimental Station of Animal Nutrition and Feed Science in the Ministry of Agriculture, Institute of Subtropical Agriculture, Chinese Academy of Sciences, Changsha 410125, China; huangpan1990@isa.ac.cn (P.H.); jianjunli@isa.ac.cn (J.L.); lcm@isa.ac.cn (C.L.)

Correspondence: nihengjia@isa.ac.cn (H.N.); yinyulong@isa.ac.cn (Y. Y.)

Table S1 Nutrition level of basal diet

Nutrient composition	Value
Protein, %	18.8
Fat, %	5.26
Fiber, %	3.06
Ash, %	5.49
Ca, %	1.12
Total P, %	0.72
SID Lys, %	1.4
SID Met + Cys, %	1.15
Total energy, kcal/g	3.61

Table S2 Composition and Nutrition level of high-fat diet

Ingredient	%	Nutrient composition	Value
Casein, 30 Mesh	25.84	Protein,%	26.2
L-Cystine	0.39	Carbohydrate,%	26.3
Maltodextrin 10	16.15	Fat, %	34.9
Sucrose	8.89	Total energy, kcal/g	5.24
Cellulose, BW200	6.46		
Soybean Oil	3.23		
Lard	31.66		
Mineral Mix	1.29		
DiCalcium Phosphate	1.68		
Calcium Carbonate	0.71		
Potassium Citrate, 1 H_2O	2.13		
Vitamin Mix V10001	1.29		
FD&C Blue Dye #1	0.26		
Total	100.00	0	

Table S3 The 16S region gene sequences of *Pedio. 6-1*, *J.Lact 12-3*, and *J.Lact 12-4*.

Bacteria	16S region gene sequences
Pedio. 6-1	TGCAAGTCGAACGAACTTCCGTTAATTGATTATGACGTGCTTGCACT<br>GAATGAGATTTTAACACGAAGTGAGTGGCGGACGGGTGAGTAACAC<br>GTGGGTAACCTGCCCAGAAGCAGGGGATAACACCTGGAAACAGATG<br>CTAATACCGTATAACAGAGAAAACCGCCTGGTTTTCTTTTAAAAGAT<br>GGCTCTGCTATCACTTCTGGATGGACCCGCGGCGCATTAGCTAGTTG<br>GTGAGGTAACGGCTCACCAAGGCGATGATGCGTAGCCGACCTGAGA<br>GGGTAATCGGCCACATTGGGACTGAGACACGGCCCAGACTCCTACG<br>GGAGGCAGCAGTAGGGAATCTTCCACAATGGACGCAAGTCTGATGG<br>AGCAACGCCGCGTGAGTGAAGAAGGGTTTCGGCTCGTAAAGCTCTG<br>TTGTTAAAGAAGAACGTGGGTGAGAGTAACTGTTCACCCAGTGACG<br>GTATTTAACCAGAAAGCCACGGCTAACTACGTGCCAGCAGCCGCGG<br>TAATACGTAGGTGGCAAGCGTTATCCGGATTTATTGGGCGTAAAGCG<br>AGCGCAGGCGGTCTTTTAAGTCTAATGTGAAAGCCTTCGGCTCAACC<br>GAAGAAGTGCATTGGAAACTGGGAGACTTGAGTGCAGAAGAGGAC<br>AGTGGAACTCCATGTGTAGCGGTGAAATGCGTAGATATATGGAAGAA<br>CACCAGTGGCGAAGGCGGCTGTCTGGTCTGTAACTGACGCTGAGGC<br>TCGAAAGCATGGGTAGCGAACAGGATTAGATACCCTGGTAGTCCATG<br>CCGTAAACGATGATTACTAAGTGTTGGAGGGTTTCCGCCCTTCAGTG<br>CTGCAGCTAACGCATTAAGTAATCCGCCTGGGGAGTACGACCGCAA<br>GGTTGAAACTCAAAAGAATTGACGGGGGCCCGCACAAGCGGTGGA<br>GCATGTGGTTTAATTCGAAGCTACGCGAAGAACCTTACCAGGTCTTG<br>ACATCTTCTGCCAACCTAAGAGATTAGGCGTTCCCTTCGGGGACAGA<br>ATGACAGGTGGTGCATGGTTGTCGTCAGCTCGTGTCGTGAGATGTTG<br>GGTTAAGTCCCGCAACGAGCGCAACCCTTATTACTAGTTGCCAGCAT<br>TCAGTTGGGCACTCTAGTGAGACTGCCGGTGACAAACCGGAGGAAG<br>GTGGGGACGACGTCAAATCATCATGCCCCTTATGACCTGGGCTACAC<br>ACGTGCTACAATGGATGGTACAACGAGTCGCGAAACCGCGAGGTTT

	AGCTAATCTCTTAAAACCATTCTCAGTTCGGACTGTAGGCTGCAACT<br>CGCCTACACGAAGTCGGAATCGCTAGTAATCGCGGATCAGCATGCCG<br>CGGTGAATACGTTCCCGGGCCTTGTACACACCGCCCGTCACACCATG<br>AGAGTTTGTAACACCCAAAGCCGGTGGGGTAACCTTTTAGGAGCTA<br>GCCGTCTAA
J.Lact 12-3	CTATACTGCAAGTCGAGCGAGCTTGCCTAGATGATTTAGTGCTTGC<br>ACTAAATGAAACTAGATACAAGCGAGCGGCGGACGGGTGAGTAACA<br>CGGGGGTAACCTGCCCAAGAGACTGGGATAACACCTGGAAACAGAT<br>GCTAATACCGGATAACAACACTAGACGCATGTCTAGAGTTTGAAAGA<br>TGGTTCTGCTATCACTCTTGGATGGACCTGCGGTGCATTAGCTAGTTG<br>GTAAGGTAACGGCTTACCAAGGCAATGATGCATAGCCGAGTTGAGA<br>GACTGATCGGCCACATTGGGACTGAGACACGGCCCAAACTCCTACG<br>GGAGGCAGCAGTAGGGAATCTTCCACAATGGACGAAAGTCTGATGG<br>AGCAACGCCGCGTGAGTGAAGAAGGGTTTCGGCTCGTAAAGCTCTG<br>TTGGTAGTGAAGAAAGATAGAGGTAGTAACTGGCCTTTATTTGACGG<br>TAATTACTTAGAAAGTCACGGCTAACTACGTGCCAGCAGCCGCGGTA<br>ATACGTAGGTGGCAAGCGTTGTCCGGATTTATTGGGCGTAAAGCGAG<br>TGCAGGCGGTTCAATAAGTCTGATGTGAAAGCCTTCGGCTCAACCG<br>GAGAATTGCATCAGAAACTGTTGAACTTGAGTGCAGAAGAGGAGAG<br>TGAACTCCATGTGTAGCGGTGGAATGCGTAGATATATGGAAGAACAC<br>CAGTGGCGAAGGCGGCTCTCTGGTCTGCAACTGACGCTGAGGCTCG<br>AAAGCATGGGTAGCGAACAGGATTAGATACCCTGGTAGTCCATGCCG<br>TAAACGATGAGTGCTAAGTGTTGGGAGGTTTCCGCCTCTCAGTGCTG<br>CAGCTAACGCATTAAGCACTCCGCCTGGGGAGTACGACCGCAAGGT<br>TGAAACTCAAAGGAATTGACGGGGGCCCGCACAAGCGGTGGAGCA<br>TGTGGTTTAATTCGAAGCAACGCGAAGAACCTTACCAGGTCTTGAC<br>ATCCAGTGCAAACCTAAGAGATTAGGTGTTCCCTTCGGGGACGCTGA<br>GACAGGTGGTGCATGGCTGTCGTCAGCTCGTGTCGTGAGATGTTGG<br>GTTAAGTCCCGCAACGAGCGCAACCCTTGTCATTAGTTGCCATCATT

	AAGTTGGGCACTCTAATGAGACTGCCGGTGACAAACCGGAGGAAG<br>GTGGGGATGACGTCAAGTCATCATGCCCCTTATGACCTGGGCTACAC<br>ACGTGCTACAATGGACGGTACAACGAGAAGCGAACCTGCGAAGGC<br>AAGCGGATCTCTTAAAGCCGTTCTCAGTTCGGACTGTAGGCTGCAAC<br>TCGCCTACACGAAGCTGGAATCGCTAGTAATCGCGGATCAGCACGCC<br>GCGGTGAATACGTTCCCGGGCCTTGTACACACCGCCCGTCACACCAT<br>GAGAGTCTGTAACACCCAAAGCCGGTGGGATAACCTTTATAGGAGTC<br>AGCCGTCTAA
J.Lact 12-4	TGCAAGTCGAGCGAGCTTGCCTAGATGATTTTGGTGCTTGCACTAAA<br>TGAAACTAGATACAAGCGAGCGGCGGACGGGTGAGTAACACGTGG<br>GTAACCTGCCCAAGAGACTGGGATAACACCTGGAAACAGATGCTAA<br>TACCGGATAACAACACTAGACGCATGTCTAGAGTTTGAAAGATGGTT<br>CTGCTATCACTCTTGGATGGACCTGCGGTGCATTAGCTAGTTGGTAA<br>GGTAACGGCTTACCAAGGCAATGATGCATAGCCGAGTTGAGAGACT<br>GATCGGCCACATTGGGACTGAGACACGGCCCAAACTCCTACGGGAG<br>GCAGCAGTAGGGAATCTTCCACAATGGACGAAAGTCTGATGGAGCA<br>ACGCCGCGTGAGTGAAGAAGGGTTTCGGCTCGTAAAGCTCTGTTGG<br>TAGTGAAGAAAGATAGAGGTAGTAACTGGCCTTTATTTGACGGTAAT<br>TACTTAGAAAGTCACGGCTAACTACGTGCCAGCAGCCGCGGTAATAC<br>GTAGGTGGCAAGCGTTGTCCGGATTTATTGGGCGTAAAGCGAGTGC<br>AGGCGGTTCAATAAGTCTGATGTGAAAGCCTTCGGCTCAACCGGAG<br>AATTGCATCAGAAACTGTTGAACTTGAGTGCAGAAGAGGAGAGTGG<br>AACTCCATGTGTAGCGGTGGAATGCGTAGATATATGGAAGAACACCA<br>GTGGCGAAGGCGGCTCTCTGGTCTGCAACTGACGCTGAGGCTCGAA<br>AGCATGGGTAGCGAACAGGATTAGATACCCTGGTAGTCCATGCCGTA<br>AACGATGAGTGCTAAGTGTTGGGAGGTTTCCGCCTCTCAGTGCTGC<br>AGCTAACGCATTAAGCACTCCGCCTGGGGAGTACGACCGCAAGGTT<br>GAAACTCAAAGGAATTGACGGGGGCCCGCACAAGCGGTGGAGCAT<br>GTGGTTTAATTCGAAGCAACGCGAAGAACCTTACCAGGTCTTGACAT

	CCAGTGCAAACCTAAGAGATTAGGTGTTCCCTTCGGGGACGCTGAG ACAGGTGGTGCATGGCTGTCGTCAGCTCGTGTCGTGAGATGTTGGGT TAAGTCCCGCAACGAGCGCAACCCTTGTCATTAGTTGCCATCATTAA GTTGGGCACTCTAATGAGACTGCCGGTGACAAACCGGAGGAAGGTG GGGATGACGTCAAGTCATCATGCCCCTTATGACCTGGGCTACACACG TGCTACAATGGACGGTACAACGAGAAGCGAACCTGCGAAGGCAAG CGGATCTCTTAAAGCCGTTCTCAGTTCGGACTGTAGGCTGCAACTCG CCTACACGAAGCTGGAATCGCTAGTAATCGCGGATCAGCACGCCGC GGTGAATACGTTCCCGGGCCTTGTACACACCGCCCGTCACACCATGA GAGTCTGTAACACCCAAAGCCGGTGGGATAACCTTTATAGGAGTCAG CCGTCTAA

Table S4 Primers used for quantitative PCR

Gene	GenBank Accession No.	Primers (5’-3’)
TNF-α	NM_013693	F: GAAAGTCAACCTCCTCTCTG<br>R: AGACTCCTCCCAGGTATATG
IL-1β	NM_008361.4	F: GAAATGCCACCTTTTGACAGTG<br>R: TGGATGCTCTCATCAGGACAG
IL-6	NM_031168	F: AGTGGCTAAGGACCAAGA<br>R: GCCGAGTAGATCTCAAAGTG
IL-10	NM_010548.2	F: TGCTAACCGACTCCTTAATGCA<br>R: TTCTCACCCAGGGAATTCAAA
TLR4	NM_021297.3	F: ATGGCATGGCTTACACACC<br>R: GAGGCCAATTTTGTCTCCACA
TLR8	NM_133212.3	F: GAAAACATGCCCCCTCAGTCA<br>R: CGTCACAAGGATAGCTTCTGGAA
β-Actin	NM_007393.5	F: GATTACTGCTCTGGCTCCTAGC<br>R: GACTCATCGTACTCCTGCTTGC
